# Answering the Big Clinical Questions in Brain Metastasis Management

**DOI:** 10.3389/fonc.2021.834122

**Published:** 2022-01-18

**Authors:** John P. Kirkpatrick

**Affiliations:** ^1^ Department of Radiation Oncology, Duke Cancer Institute, Durham, NC, United States; ^2^ Department of Neurosurgery, Duke Cancer Institute, Durham, NC, United States

**Keywords:** brain metastases, stereotactic radiosurgery, whole-brain radiotherapy, hippocampal avoidance, palliative care, leptomeningeal disease

## Abstract

Management of brain metastases is challenging, both because of the historically guarded prognosis and evolving, more efficacious treatment paradigms for metastatic cancer. This perspective addresses several of the important difficult questions that practitioners treating patients with brain tumors face in the clinic. Successfully answering these questions requires knowledge of the clinical evidence, thoughtful discussion of the patient’s goals of care and collaboration in a multi-disciplinary setting.

## Introduction

As the articles in this special issue illustrate, the management of brain metastases has changed and continues to evolve. Advances in radiation therapy, surgery and, particularly, systemic treatment of metastatic cancer have improved prognosis and increased longevity, making the preservation of neurocognition and quality of life all the more important in patients with brain metastases. The choice of treatment for brain metastases – including early and timely access to palliative care – has become more complex and complicated. At the same time, the consequences of making the optimum management choice carry higher stakes for both the patient and practitioner. While there is no single correct approach, clearly the “best” decisions will come through attentiveness to the patient’s goals of care and the input of multiple disciplines.

In this article, we share our perspective on some of the most common and important questions we encounter in the clinical management of brain metastases. The astute reader will notice that many of the “answers” to these questions, raise more questions than provide answers, and that there is no “magic eight ball” that provides a simple answer. However, a collegial effort on the part of the “village” - medical oncologists, radiation oncologists, surgeons, palliative care specialists, navigators and nurses - centered on addressing the patient’s needs and based on evidence, will provide the best care and superior outcome for the patient presenting with brain metastases.

## “My Patient Has a Large Brain Metastasis – Should I Offer Post-Op, Pre-Op or ‘No-Op’ Radiation Therapy?”

Patients often present with large brain metastases that are producing or at impending risk of causing symptomatic mass effect on brain parenchyma, critical adjacent organs and the ventricular system. These patients are typically considered for surgical resection – particularly in the case of one or two brain lesions – for rapid relief and/or prevention of mass effect and obstructive hydrocephalus. Radiation therapy is usually administered in combination following surgical resection of a brain metastasis, as either whole-brain radiation therapy (1) (WBRT) or stereotactic radiosurgery (2) (SRS) significantly and substantially reduce local recurrence compared to surgical resection alone. Post-operative SRS is frequently chosen over WBRT, given the reduced impact on neurocognition and the shorter time for recovery, which also permits more rapid initiation of systemic therapies (3).

However, even for modest-sized brain metastases, the resection cavity and resulting target volume for irradiation is often in excess of 3 cm (4), requiring a substantial dose reduction in order to administer single-fraction SRS safely (2). To overcome the limitations, radiation may be delivered over 3 to 5 fractions (hypofractionated SRS, HF-SRS), which appears to offer a better balance of treatment efficacy and toxicity (5, 6). A randomized trial of single-fraction versus hypofractionated SRS to the post-operative resection cavity is currently underway (ClinicalTrials.gov Identifier: NCT04114981.)

Pre-operative SRS is a potentially attractive alternative to post-operative SRS, as the target in the pre-operative setting is smaller and more regular with a more competent blood supply, presumably providing better oxygenation and, thus, increased radiosensitivity (7, 8). Moreover, cytoreduction of tumor by upfront SRS may reduce surgical tract contamination by viable cells during resection and consequently decrease the risk of recurrence and leptomeningeal disease (9, 10). Pre-operative SRS appears to offer very good local tumor control with minimal toxicity, and a randomized control trial of pre-operative versus post-operative SRS in brain metastases has been proposed and should provide needed information on comparative efficacy and safety of these two approaches. Note that timing of pre-operative SRS can be a challenge, particularly in the setting of symptomatic mass effect requiring immediate surgery, and the impact of up-front SRS on pathologic results is unclear.

If the patient is not surgical candidate, the treatment of large brain metastases with radiation therapy alone is an option. The need to balance toxicity with efficacy of treatment suggests that these patients may be best served by HF-SRS, as discussed above, rather than WBRT with its increased risk of neurocognitive deficits and prolonged recovery time or single-fraction SRS with greater risk of adverse-radiation effects at efficacious doses. Omitting resection in surgical candidates is more contentious. Some retrospective studies have shown substantially poorer local control for lesions exceeding 2cm diameter treated with single-fraction radiosurgery alone versus resection and radiosurgery (11), likely due to the combination of purposely reduced prescription dose for larger metastases (12) and higher tumor burden. However, other studies of single-fraction SRS using a small margin expansion about the target have not shown decreased efficacy for larger lesions (13) and studies of HF-SRS report high rates of local control for lesions >2cm diameter (6). Finally, a retrospective study of SRS alone versus resection followed by SRS found a significantly higher rate of nodular leptomeningeal disease in the surgery + SRS group versus those receiving SRS alone (21 vs 0%, P <.001) (14). This study suggests a potential advantage to avoiding surgery … or potentially utilizing pre-operative SRS.

In the absence of randomized trials of pre-op versus post-op versus no-op approaches – *which should include appropriate targeted and immunotherapy agents* – the optimal answer to the above question can best be achieved in a multi-disciplinary setting. Obviously, one needs to consider the patient’s suitability for surgery and radiosurgery, the size, location, aggregate volume and number of lesions and the patient’s performance status, disease burden and goals of care. In addition, the timing of and interaction with systemic treatments must be considered, as well as the appropriateness of any intervention in patients with poor prognosis and performance status (15). It is equally important to build a system ahead of time that can safely and adequately provide these options. For example, pre-operative SRS is only feasible when procedures for rapidly planning and delivering radiosurgery are in place, supported by robust QA processes and availability of appropriate equipment.

## “Why Would We Ever Give Whole-Brain Radiation Therapy?”

WBRT was the mainstay for the treatment of patients with multiple brain metastases for many years, providing reasonable local and distant brain control. However, WBRT produces bothersome acute toxicities in almost all patients (fatigue, scalp irritation, alopecia) and multiple studies have shown that WBRT causes significant neurocognitive deterioration versus SRS alone (16–18). Consequently, SRS and, recently, some targeted and immune therapies are emerging as the dominant treatment modality for multiple brain metastases (19). For patients with a few (≤4) brain metastases, SRS alone or in combination with surgery to the dominant lesion is often the preferred treatment, a choice somewhat obliquely endorsed by ASTRO in its “2014 Choosing Wisely” list. The indication for SRS has expanded to include larger number of brain metastases, with 10 or fewer regarded by many practitioners, as appropriate for SRS alone, based on several clinical studies coupled with advances in treatment technology. JLGK0901, a prospective observational trial, evaluated outcomes patients with 2 to 10 brain metastases treated with a multicentric, single-fraction SRS technique. The study revealed no differences in survival, local recurrence, toxicity or neurocognition in patients treated to 2-4 versus 5-10 brain metastases (20, 21). Likewise, studies in patients with 4 – 10 brain metastases treated with single-isocenter, single-fraction or HF-SRS have revealed high levels of local control with minimal neurocognitive decline (22, 23). At the same time, improved planning and treatment techniques have significantly reduced the time to treat multiple brain lesions, and there is essentially no *technical* upper limit on the number of brain metastases that can be treated with a single-isocenter intensity-modulated radiosurgery technique.

However, the technical capability to radiosurgerize 30 brain lesions should not be equated with the *clinical* appropriateness of doing so. By intent, SRS delivers a minimal dose of ionizing radiation to non-target tissue, permitting untreated sub-clinical metastases to develop into visible lesions at later date. Consequently, multiple studies show that the incidence of development of new brain metastases is far higher without than with WBRT (17, 18, 24, 25). Data from the JLK trial appear to support the assumption that the risk of microscopic disease and post-SRS distant brain disease increase with a higher number of treated brain metastases. In addition, SRS clearly does not treat diffuse leptomeningeal disease (LMD), and post-operative SRS alone has been associated with increased risk of diffuse LMD, particularly when utilized in the posterior fossa. [note that nodular LMD is not equivalent to diffuse LMD, and SRS for the former often appears to be the preferred approach (26)]. Finally, it is important to recognize that none of the published trials of multiple brain metastases randomize patients to SRS versus WBRT, and we do not know if the outcome associated with one modality is truly superior to the other (see below.) In my opinion, the patient with 25 new, small brain metastases that have appeared on a short-interval brain MRI is unlikely to realize complete control of their intracranial disease with SRS alone and it would be misleading to suggest otherwise while downplaying the value of WBRT.

In response to the above question, when the patient has a high density of brain metastases, WBRT should be considered and may be the most appropriate option *if the patient has prospects of benefitting from treatment*. As the QUARTZ study showed (15), patients with poor performance status appear to fare no better (and perhaps worse) with WBRT versus best supportive care, and the approach of palliative SRS versus supportive care alone should be considered. In patients with multiple brain metastases and a reasonable expectation of benefit from control of brain disease by WBRT, the issue then becomes effective mitigation of neurocognitive decline. While memantine and hippocampal-avoidance WBRT (HA-WBRT) utilized separately, appear to offer some benefit in reducing the depth of neurocognitive decline, the combination of the two has been shown to significantly decrease neurocognitive deterioration (27, 28). 518 patients were randomly assigned to undergo HA-WBRT plus memantine versus conventional WBRT plus memantine. Across multiple domains, HA-WBRT plus memantine better preserves cognitive function and patient-reported symptoms. While patients with lesions within 5mm of the hippocampi and diffuse LMD were excluded from the trial, it appears that patients with as few as a single metastasis were eligible and no upper limit on either number or volume of lesions was applied. It would be quite interesting to see outcome from this trial analyzed based on a stratification by number/volume of brain lesions.

Given that memantine alone offers only partial neuroprotection and that substantial changes in non-hippocampal areas of the brain are observed post HA-WBRT (29), there is interest in utilizing agents that provide more complete global protection of the brain during WBRT. For example, a novel Mn-porphyrin superoxide dismutase mimetic, BMX-001, is undergoing a randomized phase trial in patients with 5 or more brain metastases receiving WBRT.

The above discussion reflects the Author’s radiocentric experience in treating brain metastases. However, the prospect of deferring and potentially completely avoiding any radiotherapy to brain metastases is being entertained, as discussed elsewhere in this issue. For example, in non-small cell lung cancer metastatic to the brain, targeted agents with improved blood-brain barrier penetration, such as osimertinib, can effectively treat small brain metastases without brain irradiation (3, 30). However, in my experience many will eventually require brain radiotherapy. A large subset of patients brain metastases with melanoma respond quite well to dual checkpoint inhibitor immunotherapy. Tawbi et al (31) reported an “intracranial clinical benefit” (defined as the percentage of patients with complete response, partial response or stable disease at 6 months) of 57% in a cohort of 94 patients with brain metastases from melanoma treated with nivolumab + ipilimumab. The optimal combination and timing of radiation therapy, surgery and systemic treatments are poorly defined, and patients are best served by a multi-disciplinary, treatment-modality-agnostic discussion of their treatment options, preferably at a Tumor Board. It is essential that the proposed options be tailored to the patient’s tumor, performance status, overall disease state and the recommendations be thoroughly and critically discussed with the patient and their family, including the role of palliative therapy.

## “How Would You Treat My Brain Metastases if I Were Your Mother?”

As I have gotten older, this question has changed from “… if I were your mother [or father]?” to “… if I were your sister [or brother]?” Many of my colleagues would say it be more appropriate to ask “… if I were your daughter [or son]?” I now recognize that this question opens the door to an opportunity to frame the patient’s goals of care and to engage in a meaningful dialogue with the patient and their family. In a busy clinic, one’s inclination is to give the rote answer, “I treat everyone equally. I am not your relative and it would not be appropriate for me to answer that question”, moving on to a discussion of risks, benefits, side effects and logistics. However, by taking just a few more minutes at this critical point, a provider can truly help the patient chose an option best aligned with their goals of care.

If I have developed rapport with the patient, my first response is often, “well you don’t know how I feel about my mother [or father], so you may want to be careful about any answer I would give you”. This comment is surprisingly well received in most cases and is far more effective than a brief lecture on shared decision-making. Then, I typically follow-up with, “I would start by making sure I explained the different treatments to them [my parent] – as I’ve done with you – and by making sure that they *and I* understood how these options fit with their goals”. Then, either I or the patient/family member will briefly recap the patient’s goals and discuss how the management options fit with those goals. Throughout this dialogue, it is essential to repeat, acknowledge, clarify and rephrase what the patient is telling you, making liberal use of phrases, such as, “Let me make sure I’ve got this right. You want to…”

Effective multi-disciplinary management of these patients requires that all team members share the summary of these discussions with one another, with a low threshold for referral to another specialty, as needed. In particular, one must be attentive to a need for improved symptom management and home health care, areas where a Palliative Care provider can offer exceptional support to the patient and their family.

## Do I Really Need to Treat the Patient With SRS Using a Radiosurgery System?

Yes. Safe and effective SRS of brain metastases requires more than a radiosurgery capable piece of equipment. Paraphrasing the guidelines for radiosurgery proposed by Barnett et al. (32), the key elements of a radiosurgery system include:

• A multidisciplinary team consisting of a neurosurgeon, radiation oncologist and radiation physicist, all trained in radiosurgery, in general, and the specific equipment, as well as a team of dedicated radiation therapists
• Sophisticated treatment planning based on high-resolution, high-fidelity imaging that yields highly conformal, precise and accurate dose delivery to the target with minimal irradiation of normal tissues
• A linear accelerator, particle therapy unit or radioactive isotope device, capable of delivering photon or particle radiation to a remote target with better than 1 mm accuracy and precision
• A combination of patient immobilization and on-machine image guidance that ensures that the target is localized with sub-mm/sub-degree accuracy in translational/rotational accuracy
• Robust, written and rigorous quality assurance procedures for every element of the process that ensures that every element of the system is correct during each and every procedure

If these requirements cannot be met locally, an alternative approach should be considered, including referral to a radiosurgery center, use of conventional radiotherapy and/or systemic treatment with proven efficacy in treating brain metastases, as appropriate.

## Conclusion

Thoughtfully addressing the above questions with patients and their families in a multidisciplinary setting is a critical element in the treatment of brain metastases. Formulating and communicating evidence-based, specialty-agnostic recommendations with careful attention to the patient’s needs and goals of care provides the patient with the basis to make optimal, personalized decisions on their care.

## Data Availability Statement

The original contributions presented in the study are included in the article/supplementary material. Further inquiries can be directed to the corresponding author.

## Author Contributions

The author confirms being the sole contributor of this work and has approved it for publication.

## Conflict of Interest

JK has research funding from Varian Medical Systems and the BioMimetix SBIR, receives consultant fees from Monteris and is the co-owner of ClearSight RT, LLC.

## Publisher’s Note

All claims expressed in this article are solely those of the authors and do not necessarily represent those of their affiliated organizations, or those of the publisher, the editors and the reviewers. Any product that may be evaluated in this article, or claim that may be made by its manufacturer, is not guaranteed or endorsed by the publisher.
